# Editorial: Application of novel technologies for the inactivation and reduction of fungi and mycotoxins

**DOI:** 10.3389/fmicb.2025.1665940

**Published:** 2025-08-12

**Authors:** Carlos David Grande-Tovar, Gustavo Adolfo Cordero-Bueso, Clemencia Chaves-López

**Affiliations:** ^1^Grupo de Investigación Biotecnología, Facultad de Ingeniería, Universidad de San Buenaventura Cali, Cali, Colombia; ^2^Laboratorio de Microbiología, CASEM, Departamento de Biomedicina, Biotecnología y Salud Pública, Universidad de Cádiz, Cádiz, Spain; ^3^Faculty of Bioscience and Technology for Food, Agriculture and Environment, University of Teramo, Teramo, Italy

**Keywords:** mycotoxin detoxification, biocontrol, non-thermal technologies, microbial enzymes, predictive modeling

Fungal contamination and mycotoxin production continue to challenge food safety, agricultural sustainability, and public health worldwide. The vast diversity of mycotoxins, their environmental resilience, and their toxicity demand robust, interdisciplinary solutions. Recently, innovative approaches have emerged, from biological detoxification to predictive AI modeling and structure-based inhibitor design. This editorial synthesizes findings from cutting-edge studies that offer a broad, integrative view of current progress and future potential.

One of the most promising biological tools is probiotic detoxification. Murtaza et al. demonstrated the ability of *Lactobacillus plantarum* CN1 to remove up to 75.6% of zearalenone (ZEN) from dried distiller's grains and solubles (DDGS). The enhanced detoxification capacity post-autoclaving points to cell wall structural changes facilitating ZEN binding. In addition to toxin removal, the fermentation process enriched feed nutritional value, reinforcing the dual benefit of such bioprocesses.

Equally significant are yeast-based biocontrol strategies. Zhang et al. showed that xylitol supplementation boosted the antioxidative defenses and antagonistic activity of *Meyerozyma guilliermondii*, improving postharvest resistance against mold infections in apples. This work reflects a paradigm shift-leveraging metabolic priming to increase efficacy under stress conditions, thereby extending shelf-life and reducing reliance on fungicides.

To combat mycotoxins preemptively, understanding fungal ecology in processed foods is vital. Li et al. characterized fungal diversity in jujube products, revealing *Aspergillus, Penicillium*, and *Cladosporium* as dominant genera. Aflatoxin and ochratoxin contamination traced to *Aspergillus flavus* and *Aspergillus niger* highlights the need for continuous monitoring and targeted interventions during processing and storage.

Progress in biodegradation via microbial enzymes also shows strong promise. Chang et al. used *Bacillus mojavensis* L-4 in semi-solid fermentation of cornmeal to degrade ZEN, identifying key degradation enzymes and sequencing the strain's genome. Their work provides a foundation for engineering improved detoxifiers with broad-spectrum activity.

A similar direction was pursued by Subagia et al., who applied a recombinant *Bacillus subtilis* laccase (BsCotA) to detoxify aflatoxin B1 (AFB1). The codon-optimized enzyme achieved significant reduction in toxin activity (>80-fold less toxic), and the study highlighted the synergistic role of microaerobic conditions in enhancing enzyme expression and function.

Adding a novel structure-based molecular approach, Wang et al. investigated the cytochrome P450 monooxygenase AflG, a key enzyme in aflatoxin biosynthesis. Using molecular modeling, microsecond-scale molecular dynamics (MD) simulations, and high-throughput virtual screening of over 1.3 million compounds, the authors identified two potent inhibitors of AflG. These molecules disrupted AflG-averantin interactions, significantly reducing aflatoxin production in *A. flavus*. This precision-targeting strategy opens a new frontier for chemical biology in food safety: the possibility of rationally designed inhibitors to prevent toxin biosynthesis at the molecular level.

These molecular strategies are complemented by non-thermal physical methods. Molina-Hernandez et al. reviewed technologies such as high-pressure processing, pulsed electric fields, UV light, ozone, cold plasma, and blue light. These treatments offer effective fungal inactivation and mycotoxin degradation with minimal impact on food quality. However, efficacy remains dependent on fungal strain, food matrix, and specific mycotoxin structure necessitating case-by-case validation.

Among these, cold atmospheric pressure plasma (CAP) stands out. Dias de Oliveira et al. discussed how CAP's reactive species inactivate spores, disrupt membranes, and chemically modify mycotoxins like aflatoxin B1 and deoxynivalenol. CAP treatments preserve food nutrients, making them viable for large-scale postharvest use.

Finally, machine learning and AI-driven predictive models provide tools for preemptive intervention. Castano-Duque et al. used neural networks and gradient boosting to forecast aflatoxin and fumonisin risks in Illinois corn based on environmental and satellite data. With predictive accuracies up to 85% for fumonisin, such models could revolutionize monitoring and risk management systems, giving producers tools to act before contamination occurs.

Together, these studies reveal a converging future for mycotoxin mitigation where bio-based solutions, precision molecular targeting, non-thermal technologies, and predictive analytics all play synergistic roles. Future success lies in cross-disciplinary integration: microbial engineering, AI, structural biology, and food processing must work hand-in-hand.

Furthermore, regulatory adaptation will be key. As technologies like enzyme detoxification and molecular inhibitors enter the food chain, agencies must develop frameworks to assess efficacy, safety, and consumer acceptance. With supportive policy, the innovations discussed here can transform food systems from reactive containment to proactive protection.

